# The interplay of oxidative stress and immune dysfunction in Hashimoto’s thyroiditis and polycystic ovary syndrome: a comprehensive review

**DOI:** 10.3389/fimmu.2023.1211231

**Published:** 2023-07-31

**Authors:** Gabriela Batóg, Anna Dołoto, Ewelina Bąk, Iwona Piątkowska-Chmiel, Paulina Krawiec, Elżbieta Pac-Kożuchowska, Mariola Herbet

**Affiliations:** ^1^ Chair and Department of Toxicology, Faculty of Pharmacy, Medical University of Lublin, Lublin, Poland; ^2^ Department of Paediatrics and Gastroenterology, Medical University of Lublin, Lublin, Poland

**Keywords:** Hashimoto’s disease, polycystic ovary syndrome, reactive oxygen species, oxidative stress, antioxidants

## Abstract

In recent years, there has been a significant increase in the concomitant incidence of Hashimoto’s thyroiditis (HT) and polycystic ovary syndrome (PCOS), both in terms of incidence, etiology, and clinical consequences. PCOS patients suffering from autoimmune thyroid diseases show insulin resistance, impaired glucose tolerance, weight gain, and metabolic and reproductive complications. Studies have shown that chronic stress and its consequence, i.e. oxidative stress, play an important role in the pathomechanism of both disorders. It has also been shown that long-term exposure to stress triggers biological mechanisms, in particular related to the regulation of the inflammatory cascade, which plays a key role in autoimmune diseases. The paper is a review of the literature on the role of chronic stress, oxidative stress, and immune processes in the pathogenesis of HT and PCOS. In addition, the review is a source of knowledge about the treatment of these diseases, and in particular the use of antioxidants in therapeutic management.

## 1 Introduction

In recent years, there has been an increase in the incidence of Hashimoto’s thyroiditis (HT) and polycystic ovary syndrome (PCOS) in women and their coexistence (1–6). Research reveals that HT and PCOS are related both in terms of prevalence and in terms of etiology and clinical consequences, however, the exact causes have not yet been elucidated. Most published reports focus on the role of the immune system, hormonal disorders, and genetic factors. Meanwhile, it is known that chronic stress now plays a role in the pathogenesis of many diseases, including HT and PCOS (7–16). There is a known connection between chronic stress and oxidative stress, which was verified *in vivo* assays as well as in clinical trials (17–20). Surprisingly, an examination of the available literature revealed that oxidative stress, a byproduct of chronic stress, plays a significant role in the pathomechanism of both disorders (21–27). The pathomechanisms and treatment of both of these diseases separately are widely discussed in the literature, but there is no comprehensive presentation of the role of oxidative stress in the co-occurrence of these diseases, even though their co-occurrence has been noticed. Therefore, the study aims to review the literature on the role of chronic stress and oxidative stress in the pathogenesis of HT and PCOS. In addition, the review is a source of knowledge about the treatment of these diseases and, in particular, the use of antioxidants in therapeutic management.

### 1.1 Hashimoto’s thyroiditis

Hashimoto thyroiditis (HT), also known as chronic lymphocytic thyroiditis is the most widespread autoimmune disease in the world. It is characterized by lingering inflammation of the thyroid tissue and the presence of autoantibodies against thyroglobulin and thyroid peroxidase (28). In 1912 Japanese surgeon Hakaru Hashimoto (1881-1934) as a first person described this disease as a new kind of thyroid disorder with follicular inflammation, thyromegaly, and hypothyroidism (29, 30). In countries, where iodine intake is sufficient, HT is the most common cause of hypothyroidism (31). Around 20-30% of patients do not have enough thyroid hormone (32, 33). Studies showed that women are more frequently affected. The majority of them are diagnosed between the ages of 30 and 50 (34). The occurrence of antithyroid antibodies (ATA) varies with race, grows with age (35), and decreases with smoking (32, 36). The etiology of this disease is multifactorial but has not yet been fully understood. HT is thought to be caused by a combination of genetic influences, environmental risk factors, and epigenetic effects (35, 37). The aforementioned factors may influence the breakdown of immunotolerance, leading to an autoimmune attack on the thyroid (32). According to studies, pathogenic mechanisms are determined by both humoral and cellular immunity. Classically, HT was thought to be a Th1-mediated condition, but this classification has changed due to the new description of Th cell subsets. Moreover, recently it has been suggested that disturbances in the gut-thyroid axis may play a possible role in the pathogenesis of HT. Alterations in gut microbiota in HT have been found like higher diversity indices, decreased abundance of Bifidobacterium and Lactobacillus, and increased Bacteroides fragilis. Although the causal interactions between gut dysbiosis and HT are not fully elucidated, several mechanisms have been hypothesized including increased intestinal permeability and modulation of local and systemic immune response due to microbiota metabolites (38, 39).

HT is based on the lymphoid infiltration of the thyroid, including T and B lymphocytes. As a result, initially, a goiter may be formed. Hypothyroidism can develop when a significant number of follicular cells, which produce and secrete thyroxine and triiodothyronine, are destroyed. This symptom is the hallmark of Hashimoto’s thyroiditis (40). In terms of thyroid cell damage, a key role is played by cytokines that originate from the lymphocytic infiltrate. Cytokines can provoke the thyroid cells themselves to release proinflammatory mediators. In consequence, the autoimmune response is amplified and perpetuated (41). The symptoms of HT initially are not rapid and specific (**Table 1**). Depression, concentration problems, brain fog, change in mood, as well as biological changes such as hair loss, dry skin, bowel movements disturbances, constant fatigue or weight changes are a few examples (42, 43). What is more, patients can suffer from an accumulation of fluid in the pericardial cavities and pleural. This condition is called myxoedema coma. It is the most severe clinical manifestation and has to be treated as an endocrine emergency (34, 44). Due to inconclusive symptoms, the diagnosis can be troublesome. Patients’ conditions are frequently not diagnosed until late in the disease process. The most frequent laboratory results demonstrate normal to low thyroxine and elevated thyroid-stimulating hormone levels, along with increased antithyroid peroxidase antibodies. What is interesting is that there is little evidence showing the role of anti-TPO in the pathogenesis of AITD. However, so far, there has been no correlation recorded in human research between the level of anti-TPO concentration in serum and the severity of the disease (34, 45). According to statistics, anemia is present in 30–40% of patients (7). There can be reduced renal plasma flow, glomerular filtration rate, and renal free water clearance with the resulting hyponatremia. Creatine kinase is frequently increased. Prolactin levels may be elevated. Elevated total cholesterol, triglyceride, and LDL levels can occur. An ultrasound examination assesses echotexture, thyroid size, and whether thyroid nodules are present. However, it is not always required for diagnosis. Treatment should be initiated in the event of overt hypothyroidism. Patients in that condition are treated with thyroid hormone replacement therapy. Compared with untreated patients, patients treated were revealed to have a decreased risk of stroke, myocardial infarction, heart failure, cardiovascular death, and atrial fibrillation, as well as lower all-cause mortality (46–49). In recent years, there has been an increase in the incidence of Hashimoto’s thyroiditis (HT) and polycystic ovary syndrome (PCOS) in women and their coexistence (1–6). Research reveals that HT and PCOS are related both in terms of prevalence and in terms of etiology and clinical consequences, however, the exact causes have not yet been elucidated. Most published reports focus on the role of the immune system, hormonal disorders, and genetic factors. Meanwhile, it is known that chronic stress now plays a role in the pathogenesis of many diseases, including HT and PCOS (7–16). There is a known connection between chronic stress and oxidative stress, which was verified *in vivo* assays as well as in clinical trials (17–20). Surprisingly, an examination of the available literature revealed that oxidative stress, a byproduct of chronic stress, plays a significant role in the pathomechanism of both disorders (21–27). The pathomechanisms and treatment of both of these diseases separately are widely discussed in the literature, but there is no comprehensive presentation of the role of oxidative stress in the co-occurrence of these diseases, even though their co-occurrence has been noticed. Therefore, the study aims to review the literature on the role of chronic stress and oxidative stress in the pathogenesis of HT and PCOS. In addition, the review is a source of knowledge about the treatment of these diseases and, in particular, the use of antioxidants in therapeutic management.

**Table 1:** Main symptoms of Hashimoto thyroiditis and PCOS.

Symptoms of Hashimoto thyroiditis with hypothyroidism	Symptoms of PCOS
• depression • concentration problems • brain fog • change in mood • hair loss • dry skin • constipation • constant fatigue • changes in body weight • myxoedema coma	• hirsutism • weight gain • abdominal and subcutaneous fat • androgenetic alopecia • clitoromegaly • acne • insulin resistance • sleep apnea • anxiety • depression

### 1.2 Polycystic ovarian syndrome

Polycystic ovarian syndrome (PCOS) is a common endocrine disorder, that afflicts women in reproductive age. PCOS is one of the most common causes of anovulation in women (50). The morbidity rate of this disorder is 6% to 20% (51, 52). Furthermore, morbidity rates are remarkably consistent across all populations worldwide (53). The pathogenesis of PCOS has not yet been sufficiently elucidated, but some studies have shown that the occurrence of PCOS may be closely related to metabolic, genetic, immunological, and hormonal factors (54).

Hormonal dysfunctions play an important role in the etiology of PCOS. The cause of this disease is the dysregulation of female sex hormones, which leads to cysts in the ovarian antral follicles. Rotterdam criteria are used to diagnose PCOS. It requires two of the following three characteristics to be present: oligo- or anovulation, clinical or biological hyperandrogenism, and polycystic ovaries (55). A cyst is a sac filled with water and an egg, which is typically discharged for potential fertilization. A functional cyst, which is the result of the conversion of an egg into the cyst, inhibits ovulation. Inhibition of ovulation induces dysfunction of the menstrual cycle, causing amenorrhea. The presence of multiple cysts in the ovarian follicles is characterized as PCOS. In normal conditions, ovarian theca cells provide support to developing follicles in their formation of mature oocytes. In PCOS, theca cells are extra responsive to the stimulation of insulin, whereupon they proliferate and cause ovarian hyperthecosis (56). Insulin resistance is a significant dimension in PCOS, because it increases androgenic potential in theca cells, exacerbating PCOS. Theca cells are very sensitive to the gonadal steroid gonadotropin, which cause hyperandrogenism in PCOS patients (56). Moreover, in cultured conditions, human theca internal cells, that were removed from PCOS ovaries indicate elevated androgen secretion, which persisted during long-standing culture (57). Elevated androgen level leads to anovulation (58). The dysfunctional secretion of gonadotropin-releasing hormone (GnRH) from the hypothalamus is a significant factor in the etiology of PCOS. GnRH stimulates the secretion of follicle-stimulating hormone (FSH) and luteinizing hormone (LH) from the pituitary gland. These hormones are indispensable for the proper progression of two dissimilar phases of the menstrual cycle. In PCOS, the concentration of FSH and LH hormones is insufficient. As a result, the egg is not formed or cannot be liberated from the follicle. Furthermore, high levels of prolactin can inhibit GnRH hormone production. Androgens are produced by some of the cysts. Female virilization and male appearance are caused by hyperandrogenism. Hirsutism, weight gain, abdominal and subcutaneous fat, androgenetic alopecia, clitoromegaly, and acne are all visible signs of hyperandrogenism (56). Except for noticeable changes, abnormalities in the basic metabolic panel are presented. Insulin resistance is very common in PCOS. The result of insulin resistance may be hyperinsulinemia and, next, diabetes mellitus. Hyperinsulinemia causes abdominal obesity or central obesity. In PCOS patients’ dyslipidemia, hypertension, and cardiovascular issues are common morbidities (56). Sleep apnea, which involves 20% of PCOS patients, is another sign, that is caused by elevated steroid hormone levels (56, 58). Psychiatric symptoms, such as anxiety, and depression, are also present in PCOS (**Table 1**) (58). Many factors are causing PCOS. Thyroid disorders, Cushing’s syndrome, hyperprolactinemia, androgen-secreting tumors, and congenital adrenal hyperplasia can lead to PCOS. In the development of the PCOS, exposure to chemicals is a significant factor. In cosmetics, there are many chemical compounds, such as phthalates, parabens, benzophenones, metals, and glutaraldehyde, which are endocrine disruptors (56). Genetic predispositions are also a risk factor for PCOS (5, 56). A growing body of evidence suggests that gut microbiota may also play a role in the development of PCOS. Changes in gut microbiota composition in patients with PCOS included a higher abundance of *Lactobacillus, Escherichia/Shigella*, and *Bacteroides.* Gut dysbiosis may affect the metabolism of sex hormones and insulin, follicular development, and regulation of the immune system, potentially participating in the development of PCOS (59–61).

## 2 Correlation between PCOS and HT

Based on available publications, the correlation between PCOS and HT is evident. In PCOS patients, the prevalence of Hashimoto’s thyroiditis is higher than in women without this disease. Also, in patients with HT the prevalence of PCOS is higher than in patients without HT (6). The risk of HT in PCOS is 3.27 times higher in Europe (62). According to Chuan-Wei Ho the PCOS risk in Taiwanese women with HT is 2.37 times higher. The risk of HT in Asian PCOS patients increases by 4.56 times (62). We do not know if PCOS influences the development of HT or if HT causes the development of PCOS (5). Ovarian destruction may be caused by greater exposure to ovaries autoantibodies. Similar processes are involved with the thyroid gland in HT (63). The result of both disorders may be insulin resistance, impaired glucose tolerance, and weight gain (5, 6). What is more, these disorders are causes of infertility. The presence of both of these disorders may result in more serious metabolic and reproductive complications than just one. PCOS patients who suffer from thyroid autoimmune disease exhibit greater susceptibility to infertility. Despite the well-documented genetic influence on this disorder, the mechanism of PCOS and HT is unclear (5). However, low-grade chronic inflammation is known to play an important role in promoting the occurrence and development of PCOS and is also associated with the occurrence of HT (64, 65). Advanced glycosylation end products (AGEs) and their receptors are overexpressed in women with PCOS in inflammatory stress cascades (66). Studies have shown that adipose tissue plays an important role as a pro-inflammatory factor in the pathogenesis of PCOS, and the release of tumor necrosis factor alfa (TNF-α) and interleukin-6 (IL-6) from macrophages is associated with the induction of insulin resistance (67). Autoimmune antibodies, including anti-smooth muscle antibodies, anti-nuclear antibodies, anti-histone antibodies, and anti-double-stranded DNA (dsDNA) antibodies are also increased in PCOS patients (68). Thus, the above evidence points to a relationship between HT and PCOS and reveals common mechanisms indirectly related to inflammatory processes. Moreover, there is substantial evidence that inflammatory processes play a key role in the relationship between exposure to chronic life stress and are linked to many diseases, including insulin insensitivity (69). It has been revealed that prolonged exposure to stress triggers biological mechanisms, in particular those related to the regulation of the inflammatory cascade, which plays a key role in the pathogenesis of many diseases. In HT, immune antibodies damage thyroid cells, resulting in the development of inflammation. (TNF-α), interleukin (IL)-1α, IL-6, IL-8, IL-10, IL-12, and interferon-γ (IFNγ) are produced by thyroid follicular cells and intra-thyroidal inflammatory cells. Furthermore, in hypothyroidism, the stimulatory effect of hormones on the above-mentioned cytokines is responsible for the increase in IL-12 and IL-2 synthesis (70–73). Similar observations apply to patients suffering from PCOS (67). In the case of PCOS, the inflammatory process is associated with adipose tissue, resulting in elevated levels of inflammatory factors such as TNF-α, IFNγ, and Il-6. Obesity and insulin resistance also play a role in the inflammatory process taking place in the body of affected women. Elevated leptin levels, which are observed in obesity, increase the production of pro-inflammatory cytokines such as TNF-α, IL-6, and IL-12 (52, 64, 67, 74, 75). Therefore, it seems very important to take these aspects into account in the coexistence of HT and PCOS.

## 3 Chronic stress in HT and PCOS disease

The tests on animals and humans show that chronic stress leads to many autoimmunological diseases due to its destructive affection on the immune system and its responses. It also has a huge impact on the endocrine system which manifests itself as malfunctioning endocrine glands and leads to endocrine disorders (7). Stressful situations lead to the activation of the stress cascade. Activation of this type of reaction influences the nervous system, immune system, and endocrine system, and it also interferes with internal homeostasis. The brain gives the signal directly to the immune system. It also receives signals *via* the hormonal system and the nervous system *via* indirect pathways. The main components of the stress cascade are corticotropin and the releasing corticotropin-releasing hormone (CRH), which are localized in the hypothalamus. Important parts of that cascade are also noradrenergic neurons located in the brainstem and their corresponding peripheral effectors. This group includes the axis of the pituitary and the adrenal glands, as well as the sympathetic nerves. There is a very important bond between the hypothalamic-pituitary-adrenal (HPA) axis and the sympathoadrenal system that allows them to interact with each other. During the stress reaction, the brain uses a variety of relays to activate the nervous system, hormonal system, and immune system. Direct agitation of the immune system by the brain and indirect agitation by the nervous and hormonal systems lead to the disruption of homeostasis and the development of autoimmunity (8). The influence of the HPA axis on the immune response can occur through three mechanisms involving the influence of hormones of adrenocorticotropic origin, the influence of hypothalamic hormones, and the activity of pituitary hormones. Mechanism one is based on the inhibitory effect of glucocorticosteroids on immune helper cells and leukocytes. These hormones suppress the activity of these cells and reduce the synthesis of cytokines and mediators involved in the induction of inflammation. In addition, they have an inhibitory effect on the activity and function of Th1 lymphocytes while inducing apoptotic processes in individual T cells and eosinophils. In addition, through their anti-inflammatory effect, they cause restrictions in the synthesis of IL-6 and IL-1b. By affecting enzymes, these hormones lead to a reduction in the synthesis of prostanoids, nitric oxide, or platelet-activating factors. The second pathway of influence of the HPA axis is based on the action of the pituitary hormones beta-endorphin and corticotropin, which have pro-inflammatory as well as immunopotentiating effects. Beta-endorphin synthesized at inflammatory sites shows strong pain-suppressing properties, but the exact pro-inflammatory and immunomodulatory mechanisms of both compounds in ongoing inflammatory processes are not fully understood. The last mechanism is related to the pro-inflammatory effects of CRH and the potential pro-inflammatory properties of arginine vasopressin. Increased concentrations of CRH and products of its metabolism are observed at sites of ongoing active inflammation. High levels of this molecule are detected in the synovial fluid examined in patients suffering from rheumatoid arthritis and also in those affected by HT (76). Available publications report on CRH’s ability to enhance proliferation and expression toward IL-2 receptors and on its ability to stimulate T and B lymphocyte proliferation processes. The induced processes secondarily lead to the intensification of lysis involving NK cells, synthesis and release of IL6, IL1, and IL2, as well as suppression of interferon-gamma synthesis (77). A prolonged pathological inflammatory response can lead to the induction of disturbances in the redox profile of cells and, consequently, to the induction of a state of oxidative stress. Cells involved and stimulated to eliminate the induced inflammatory process in their activity lead to the synthesis of hydrogen peroxide and hydrogen peroxide categorized as ROS. The overall stimulation of immune cells continues until the inflammation is eliminated so that chronic inflammation caused by several etiological factors can contribute to the excessive production of ROS, subsequently causing a state of oxidative stress in the cells affected by inflammation (78). The influence of the HPA axis on the immune response can occur through three mechanisms involving the influence of hormones of adrenocorticotropic origin, the influence of hypothalamic hormones, and the activity of pituitary hormones. Mechanism one is based on the inhibitory effect of glucocorticosteroids on immune helper cells and leukocytes. These hormones suppress the activity of these cells and reduce the synthesis of cytokines and mediators involved in the induction of inflammation. In addition, they have an inhibitory effect on the activity and function of Th1 lymphocytes while inducing apoptotic processes in individual T cells and eosinophils. In addition, through their anti-inflammatory effect, they cause restrictions in the synthesis of IL-6 and IL-1b. By affecting enzymes, these hormones lead to a reduction in the synthesis of prostanoids, nitric oxide, or platelet-activating factors. The second pathway of influence of the HPA axis is based on the action of the pituitary hormones beta-endorphin and corticotropin, which have pro-inflammatory as well as immunopotentiating effects. Beta-endorphin synthesized at inflammatory sites shows strong pain-suppressing properties, but the exact pro-inflammatory and immunomodulatory mechanisms of both compounds in ongoing inflammatory processes are not fully understood. The last mechanism is related to the pro-inflammatory effects of CRH and the potential pro-inflammatory properties of arginine vasopressin. Increased concentrations of CRH and products of its metabolism are observed at sites of ongoing active inflammation. High levels of this molecule are detected in the synovial fluid examined in patients suffering from rheumatoid arthritis and also in those affected by HT (76). Available publications report on CRH’s ability to enhance proliferation and expression toward IL-2 receptors and on its ability to stimulate T and B lymphocyte proliferation processes. The induced processes secondarily lead to the intensification of lysis involving NK cells, synthesis and release of IL6, IL1, and IL2, as well as suppression of interferon-gamma synthesis (77). A prolonged pathological inflammatory response can lead to the induction of disturbances in the redox profile of cells and, consequently, to the induction of a state of oxidative stress. Cells involved and stimulated to eliminate the induced inflammatory process in their activity lead to the synthesis of hydrogen peroxide and hydrogen peroxide categorized as ROS. The overall stimulation of immune cells continues until the inflammation is eliminated so that chronic inflammation caused by several etiological factors can contribute to the excessive production of ROS, subsequently causing a state of oxidative stress in the cells affected by inflammation (78).

The stress reaction activates the HPA-axis and also the sympathoadrenal system. This entails an increase in the release of stress hormones like glucocorticoids and catecholamines (9, 27). Those stress hormones can interact with antigen-presenting cells and have an impact on the direction of T-helper lymphocyte differentiation. This situation can lead to a shift in the balance of T helper lymphocyte differentiation toward Th2 rather than Th1, which manifests itself as an increase in humoral immunity and a decrease in cellular immunity. An enhanced immune response involving Th1 lymphocytes can be devastating to the thyroid gland by inducing apoptotic processes in follicular cells. This results in the destruction of gland cells and the development of inflammation (8, 24). Stress also affects the functioning of the hypothalamic-pituitary-thyroid axis. As a result, the secretion of thyroid-stimulating hormone (TSH) by the pituitary gland is reduced, despite the normal daily rhythm of hormone secretion being maintained. There is an impaired conversion of thyroxine to triiodothyronine in the body and an overall reduction of the TSH feedback to the thyrotropin-releasing hormone (9).

Polycystic ovary syndrome is another disease entity in which stress plays a role in the pathogenesis (10, 79). Chronic stress contributes to pathological polycystic ovary syndrome by overstimulating the hypothalamic-pituitary-adrenal axis and by increasing stimulation and activity of the sympathetic nerve of the ovary (79). Studies on rats also show that 12 weeks of exposure to chronic stress leads to elevations in serum substances such as insulin and corticosterone. An increase in fasting blood glucose is also observed. Along with these lesions, cystic follicles were also observed. This study shows that chronic stress leads to elevated levels of glucocorticoid which entail the development of hyperglycemia. Long-term elevated glucose levels in the body lead to the development of insulin resistance and subsequent hyperinsulinemia, which may lead to increased androgen synthesis by ovarian theca cells. The processes listed above induced the development of polycystic ovary syndrome in the female rats in this study (23). Disturbances in the normal functioning of the HPA axis and the increased cortisol levels associated with them also contribute to the pathogenesis of this condition (11). It has been found that chronic stress may induce inflammation of the hypothalamus, and may lead to increased activation of the HPA axis and its dysregulation, which in turn may lead to endocrine and reproductive disorders (80). Glucocorticoids play a major role in stress-induced hypothalamic inflammation. These hormones play an important role in the regulation of the HPA axis, which involves a very large number of receptors for glucocorticoids located in the hypothalamus. When the stress axis is activated, elevations of these hormones are observed. Chronic stress causes the development of glucocorticoid resistance and leads to the inhibition of normal negative feedback on the hypothalamic–pituitary–adrenal axis. This can lead to altered normal tissue responses to glucocorticoids. All these processes lead to the expression of the Iba1 protein, causing the production of inflammatory molecules and sensitization to other stimuli. Increased synthesis of interleukin-1, stimulating the HPA axis, and interleukin-6 are also observed. As a result of the intensification of the above-mentioned processes in the body during chronic stress, a vicious circle of activation of the HPA axis develops, which may lead to the development of hypothalamic inflammation (79).

## 4 Connection of chronic stress and oxidative stress

Oxidative stress is a condition of the body in which there is a significantly increased concentration of reactive oxygen species relative to antioxidant substances (18, 81–84). Molecules called reactive oxygen species are naturally produced by mitochondria (83, 85). The synthesis of these compounds is the result of a normal metabolic pathway (82, 83). In a physiological state, these molecules are used as substrates to carry out essential metabolic reactions (18) or act as signaling molecules (82). The literature also reports that they are essential substances involved in the proper function and development of nerve cells (86). When there is an imbalance in the body between the intensity of the synthesis of reactive oxygen species and the mechanisms responsible for antioxidation, oxidative stress occurs (18, 84).

There are two sources of reactive oxygen species: endogenous and exogenous (84, 85). Mitochondria and peroxisomes are two major groups of cellular organelles responsible for the endogenous production of reactive oxygen species. Reactive oxygen species produced by mitochondria are mainly formed during oxidative phosphorylation. Mitochondrial complexes III and I are mainly involved in the synthesis of these molecules. The result of the work of these structures is the formation of superoxides. Electron chain transport also plays a significant role. Peroxisomal synthesis mainly involves hydrogen peroxide and hydrogen peroxide. The enzymes involved in the synthesis are mainly peroxisomal xanthine oxidase and peroxisomal acyl-CoA oxidase. Another class of cells involved in the synthesis of reactive oxygen species are inflammatory cells such as macrophages and neutrophils. Under physiological conditions, these cells use these compounds to neutralize bacterial cells. The synthesis of reactive oxygen species with the involvement of inflammatory response cells is carried out by enzymes such as NADPH oxidase. In an agitated state, these cells increase oxygen uptake. This stimulates NADPH oxidase to synthesize reactive oxygen species. This results in the synthesis of hydrogen peroxide, which can be further metabolized by superoxide dismutase (76, 78). Chemical compounds foreign to the body (xenobiotics) and ionizing radiation are mainly responsible for the exogenous synthesis of reactive oxygen species. Water radiolysis is a major pathway for the synthesis of reactive oxygen species initiated by ionizing radiation. Through the synthesis of these molecules, gene mutation, damage to the cell’s nucleic acids, and the death of the cell can occur. These actions result in distant and acute toxic effects. Xenobiotics that are involved in the exogenous synthesis of reactive oxygen species include barbiturates, chlorinated compounds, phorbol esters, ions of certain metals, and peroxisome proliferating compounds (85). Some sources also state that exogenous sources also include tobacco, industrial solvents, selected drugs (gentamicin, tacrolimus, and bleomycin), alcohol, water and air pollution, and food processing such as smoking meat (84). It is suspected that these compounds can stimulate the synthesis of reactive oxygen species in two ways: by stimulating an endogenous source or by transforming the compound (85).

The main components of oxidative stress are reactive oxygen species and reactive nitrogen species (84). The main pool of reactive oxygen and nitrogen species is free radicals. These are molecules characterized by very high reactivity and the ability to damage cellular structures. Free radicals are capable of initiating these changes due to the presence of unpaired electrons in their structure (84, 87). Reactive oxygen species that play the greatest role in the initiation of oxidative stress include hydrogen peroxide, superoxide anion radical, and hydroxyl radical (82). H2O2, which is not a radical but has high reactivity, also plays a significant role (87). The family of reactive nitrogen species that play an important role in the initiation of oxidative stress includes nitrate, nitrogen dioxide, nitrite, nitric oxide, and peroxynitrite. Reactive sulfate and reactive carbonyl forms also play a minor role. The former group includes methionine forms, cysteine forms, and selected compounds such as mycothiol, glutathione, or trypanothione, which are characterized by low molecular weight. Group two is defined by excited carbonyl moieties and selected types of aldehydes (88). Oxidative stress has a detrimental effect on nucleic acids, lipids, and proteins, disrupting their normal structure and function (83, 85, 86, 88, 89). By interacting with DNA, these compounds lead to the modification of the bases. These changes lead to deletions, translocations, point mutations, or insertions. They also show toxic effects on mitochondrial DNA, which is sensitive to such compounds. As a result of the effects of reactive oxygen and nitrogen species on these structures, modifications occur in the structure and function of various proteins. These modifications can also be seen in the disruption of normal gene expression (85, 90). These compounds also interact with cellular RNA, leading to the breaking of nucleic acid strands, the excision of bases in the chain, and the substitution of the correct bases. All of these changes in the normal RNA chain can lead to disruption of normal gene expression or adverse changes in protein synthesis (85). Proteins are another group of cellular organelles whose proper function and function are influenced by oxidative stress. It interferes with the normal nitration and carbonylation of phenylalanine and tyrosine groups of proteins. These changes can manifest themselves in abnormal cross-linking of proteins, disrupt the normal structure of bonds between proteins, or lead to protein degradation. The effect of these changes is to alter the function of enzyme proteins involved in DNA repair. Receptor and transporter proteins may also be modified, or normal protein metabolism may be halted (85). Peroxidation is the effect of reactive oxygen and nitrogen species compounds on lipids (85, 91). Sources say that the compounds responsible for creating oxidative stress affect bio-membranes (91), modify the normal structure and function of cell organelles and cell membranes, and initiate the synthesis of genotoxic compounds that can interfere with DNA leading to mutations. In addition, the synthesis of lipid hydroperoxides and lipid peroxyl radicals can occur as a result of oxidative effects on polyunsaturated fatty acids (85). Moreover, the metabolism of compounds produced by lipid peroxidation results in the formation of various types of prostaglandin-like structures and aldehydes, which are highly reactive and capable of reacting with DNA (forming adducts) (85).

Many studies confirm the correlation between exposure to chronic stress and the occurrence of oxidative stress and elevated markers of oxidative stress (17, 18). The literature shows that chronic stress carries an increased risk of oxidative stress (17, 18). In studies, it has been proven that high doses of cortisol administered over a long period of time drastically reduce normal mitochondrial function and increase cell death pathways (18). To link the effects of chronic stress on the body and the induction of oxidative stress, a study involving 48 women was performed (18). The women in the study were post-menopausal, and involved in the care of husbands with dementia; the control group was made up of women whose husbands did not have dementia. The study evaluated the levels of markers characteristic of oxidative DNA and RNA damage (8-hydroxyguanosine, 8-iso-prostaglandin, 8-hydroxy-20-deoxyguanosine). During the study, the subjects were exposed to laboratory stress. During this event, a saliva sample was taken from them to assess their cortisol levels. The study showed that the female participants subjected to chronic stress had significantly elevated concentrations of the oxidative stress marker 8-hydroxyguanosine (18).

Studies using animals show that the long-term effects of cortisol can affect individual markers of oxidative stress (19, 20, 92–94). A study conducted on rats proved that cortisol administration increased oxidative damage to DNA material, but did not lead to an increase in the rate of production of reactive oxygen species in the liver (19, 94). The study was conducted using 30 male rats. There were 10 test individuals in each group. For the study period of 4 weeks, corticosterone was administered to the rats in doses along with their food: 0mg (control group), 150 mg (group II) and 400 mg (group III)/kg of food. The animals were then sacrificed 4 weeks after the start of the study, and their livers were harvested to evaluate oxidative stress-related indicators. The extent of oxidative stress-induced protein and mtDNA damage was examined, and fatty acid analysis was performed. H2O2 synthesis and oxygen consumption by mitochondria were also assessed. After measurements were made, it was proven that none of the doses of corticosterone administered significantly increased the synthesis of H2O2 by mitochondria. Also, oxygen consumption in the groups of animals given corticosterone did not increase. A shortening of fatty acid chain lengths was observed in both groups. Compared to the group that was not given corticosterone, the level of the mtDNA oxidative damage marker (8-oxodG) reached considerably higher values (19).

Another animal study evaluated the effects of chronic stress induced by immobilizing an animal for 21 days on the activity of oxidative stress-related indicators. Levels of glutathione peroxidase (GPx), superoxide dismutase (SOD), glutathione reductase (GR), malondialdehyde (MDA), reduced glutathione (GSH), and catalase (CAT) were measured. The study was carried out to evaluate the ability of carnose to protect against the adverse effects of oxidative stress. Nine groups of test animals were formed, with eight individuals randomly assigned to each group. To induce chronic stress, the test animals were placed in special restraints. During the study period, the rodents were housed there for 21 days at a rate of 1 hour/day. The study found that rodents subjected to chronic stress by immobilization showed elevated levels of total corticosterone and increased levels of the lipid peroxidation index (MDA). In parallel with these changes, a decrease in the activity of antioxidant enzymes (GPx, CAT, GR, SOD) and GSH was noted (20).

### 4.1 Oxidative stress in HT

There are a growing number of reports showing that increased oxidative stress pathways lead to increased synthesis and release of a wide variety of immune and inflammatory molecules. These molecules can often lead to damage to many tissues (27, 95, 96). Studies have shown that oxidative stress affects the development of hypothyroidism. In this condition, a deficiency of antioxidant-like substances leads to oxidative stress in cells (25, 26). In Hashimoto’s disease, significantly reduced overall antioxidant status, increased lipid peroxidation, and increased overall oxidative status have been observed (95). T and B lymphocytes play a significant role in this disease. In pathological situations, these molecules can contribute to increased oxidative stress pathways and the over-synthesis of reactive oxygen species. The mechanism is based on enzymes such as nicotinamide adenine dinucleotide phosphate oxidase. Excessive activation of B and T lymphocytes leads to a significant increase in total hydrogen peroxide concentration, which will further lead to thyrocyte necrosis and apoptosis (25). A state arises in which the resulting free radicals cause damage to nucleic acids, carbohydrates, and cell-building lipids and proteins (25, 27). All of the aforementioned processes lead to the destruction and necrosis of thyrocytes which is caused by a disturbed oxidative state. Another correlation between Hashimoto’s disease and oxidative stress is a noticeable reduction in the superoxide dismutase enzyme (25). Also, it’s been shown that excessively high levels of the hormone TSH lead to increased pathways of oxidative stress and its induction. Lipid peroxidation, increased protein oxidation, and increased carbonyl concentration have all been observed (26). Studies show that levels of oxidative stress are correlated with the presence of anti-thyroperoxidase antibodies. Patients with these antibodies show increased oxidative stress pathways compared to the results of patients without these antibodies (26). To demonstrate the correlation between the severity of the oxidative stress system, and the severity of hypothyroidism, a study was conducted involving 20 patients with hypothyroidism of Hashimoto’s syndrome type (30). Metabolites and substances such as ascorbate, superoxide dismutase, malondialdehyde, myeloperoxidase, and nitrites were measured in the fasting blood of patients. Vitamin A, E, and β-carotene levels were also measured. The conclusion of the study presented a disruption of the body’s antioxidant system and an increase in reactive oxygen species (ROS) synthesis in patients suffering from hypothyroidism. ROS, which belongs to oxygen-free radicals, are provided by the reduction of molecular oxygen and produce side effects of metabolism and aerobic respiration. They were observed to have increased levels of nitrite, myeloperoxidase, and malondialdehyde. Vitamin E levels also increased (28). Another study shows a connection between Hashimoto’s type of hypothyroidism and the presence of anti-thyroid antibodies and the presence of an oxidative stress state and its indicators (25). The patients studied included cases of overt hypothyroidism, euthyroidism, and subclinical hypothyroidism. A total of 124 people participated in the survey. The group included both sick (93) and healthy (31) people. The study lasted one year (September 2013 – September 2014) and was conducted using fasting venous blood. The study showed an increase in the oxidative stress index and an increase in the total oxidative stress index in those diagnosed with overt hypothyroidism. A decrease in aryloesterase enzyme, thiol, and antioxidant status was also noted in this group. Levels of indicators associated with the existence of oxidative stress increased in each of the subpopulations of Hashimoto’s disease patients studied. This study showed an inverse relationship between the level of total antioxidant status and antithyroid peroxidase and paraoxonase. A similar relationship was noted between the concentration of total thiol and the concentration of antiglobulin (25). Another study shows a link between the euthyroid hypothyroidism of Hashimoto’s type and overall oxidative balance (27). The study is based on the measurement of specific markers of oxidative stress: biological antioxidant potential and reactive oxygen metabolites. Specific protein oxidation products and the level of glycation end products were also taken into consideration in evaluating the final results. The experiment was conducted on 134 subjects (71 patients and 63 healthy subjects). Elevated levels of reactive oxygen metabolites and glycation end products have been observed in patients with Hashimoto’s disease. In addition, the level of antioxidant potential was reduced in these individuals. This study confirmed increased oxidative stress pathways and an oxidative reduction imbalance in patients with Hashimoto’s disease. A significant increase in the concentration of oxidants suggests a clear intensification of the oxidative system and weakening of the antioxidant system (27).

### 4.2 Oxidative stress in PCOS

Oxidative stress plays a very important role in the etiology of PCOS. There are known numerous studies about the level of oxidative and antioxidative markers; in PCOS patients oxidants-antioxidants imbalance is present (21, 97). It is caused by concurrent this disease metabolic afflictions, like hyperinsulinemia, obesity, and dyslipidemia. Each of these afflictions is a contributor to oxidative stress. Obesity leads to lipid catabolism and consequently to stirring free radicals. Hyperglycemia escalates the amount of ROS, which is engendered by mononuclear cells. The rising level of the inflammatory transcription factor, nuclear factor-kappa B, and activation of TNF-α is caused by ROS. Inflammatory markers cause hyperandrogenism by stimulating the production of androgens in ovaries and in this way associate inflammation with PCOS. The implications of the occurrent process are the formation of cysts in ovaries, decrease ovarian follicles quality, and infertility (21). Available studies show dysfunctions in the level of inflammatory markers and antioxidant enzymes in patients with PCOS. Based on these studies, there is seen a correlation between oxidative stress, chronic inflammation, and morbidity for PCOS (21). In one of the available studies, the indicators of oxidative stress have been measured. Patients have been divided into three groups: patients with PCOS, patients with PCOS and metabolic syndrome (MS), and healthy patients (control group). Malondialdehyde was marked as an oxidative indicator and superoxide dismutase (SOD), total antioxidant activity (TAA), vitamin C, vitamin E, and retinol were marked as antioxidative indicators. In the group with PCOS and the group with PCOS and MS, the level of MDA was higher than in the healthy patient group. The level of antioxidative indicators was lower in the PCOS without and with MS group concerning the control group. The highest imbalance between oxidative indicators and antioxidative indicators occurred in the group with PCOS and MS (23).

In another study, patients were divided into two groups: with PCOS and without PCOS (control group) (24). Both groups were marked (GPx). GPx takes part in the reduction of H2O2 and lipid peroxides to water and lipid alcohols and the oxidation of GSH to GSSG. In PCOS patients’ level of GPx was higher than in the control group. Another marked indicator was glutathione reductase (GR), which is a catalyzer reduction GSSG to GSH. In PCOS patients’ level of GR was higher than in the control group. A decrease in GSH levels was observed in PCOS patients. GSH plays a crucial role in protecting cells against oxidative damage. Higher ROS formation is caused by greater androgen production. GSH is used in bigger quantities to protect cells against damage, and conseqently decrease in GSH is observed. TAC (total antioxidant capacity) was the last antioxidant indicator, that was marked in this study. TAC determines the antioxidant potential of body fluids. Its level was lower in the PCOS patients group (24).

## 5 Treatment of HT

According to the study by Vickery and Hamlin, many patients do not require treatment, as the disease is often asymptomatic and the goiter is small (98). They revealed that it is possible the disease will remain stable for many years and that the clinical condition will not change. It was proven on clinical and pathologic grounds. When goiter is a problem as a result of local pressure symptoms, thyroid hormone therapy is indicated (99). After several months of treatment, this therapy often causes a satisfying reduction in the measurement of the goiter. In young patients, the results are especially impressive. A full replacement dose is indicated if hypothyroidism is present. In some cases, the sudden onset of the disease, combined with pain, has required therapy with glucocorticoids. In some studies, it has been revealed that glucocorticoids increase plasma T4 and T3 levels by delaying the autoimmune process (100). Treatment with chloroquine or X-ray therapy is not advised because of its toxicity and severe side effects (101, 102). The immune-mediated disorder requires a diet that supports the immune system in regulating inflammatory processes through meals in terms of their ingredients and preparation, and also the elimination of problematic food antigens (42). Antioxidants such as iodine, zinc, selenium, natural polyphenols and alkaloids, inositol, L-carnitine, and probiotics are important in the treatment of HT (103). In addition, monounsaturated fatty acids (MUFA), such as oleic acid, reduce ROS production and protect against oxidative stress. A strong antioxidant effect is also shown by resveratrol, curcumin, and berberine - their effect on reducing the production of ROS, oxidative stress, and anti-inflammatory activity has been confirmed in many studies (103). Treatment with chloroquine or X-ray therapy is not advised because of its toxicity and severe side effects (101, 102). According to recent research supplementation with Selenium (Se) affects Hashimoto’s thyroiditis (104). According to the findings of the study, Se has the potential to lower TSH levels in the development of Hashimoto’s thyroiditis and thyroid antibodies. Selenium is an essential trace mineral because of its anti-inflammatory and antioxidant features. Selenium occurs in specific selenoproteins such as selenocysteine (105). The main selenoproteins, including glutathione peroxidase, thioredoxin reductases, and deiodinases, are expressed in the thyroid in large quantities. The primary function of glutathione peroxidases is to protect the body from damage caused by oxygen free radicals, and each enzyme has a specific location (105, 106). Thyroid hormone synthesis needs thyroglobulin iodination by thyroperoxidase in the presence of hydrogen peroxide. Potentially dangerous for thyrocytes, the synthesis of H2O2, is regulated by TSH by a complex system of secondary relays and seems to be the stage limit for the synthesis of thyroid hormone when sufficient iodine is available. Such an organization allows the H2O2 created at the surface of thyrocytes to be quickly used for the reaction of iodination, while the intracellular H2O2 is reduced by antioxidant enzymes such as TRs, GPXs, and catalases (107, 108). In pigs, a lack of selenium causes a decrease in GPX intracellular activity, which leads to protein cytoplasmic iodination after H2O2 exposure; however, when sufficient selenium is available, the iodination reaction is limited. Apoptosis is induced in human thyroid follicle cultures by high doses of d’H2O2, TGF-, or iodine. Preincubation with low doses of selenium escalates the activity of GPX and reduces cell death (109). Selenium intake may also protect against autoimmune thyroiditis, although the results have been ambiguous (104). Zinc is another necessary micronutrient for thyroid function. It is required to catalyze the T4-to-T3 conversion reaction. Superoxide dismutase enzyme includes zinc, which is considered antioxidative. Furthermore, zinc is an element of the thyroid hormone-binding transcription factor. Zinc insufficiency affects the thyroid on many levels: it impairs TRH synthesis, but also TSH, T4, and T3 (110–114). In studies with rats, a zinc shortage reduced free levels of T4 and T3 by around 30% (115). In humans with a zinc shortage, TSH, T3, and T4 decrease as well, and patients with hypothyroidism often have decreased levels of zinc and copper (116). Iodine is necessary for the synthesis of thyroid hormones (117, 118). The relationship between iodine intake and the presence of circulating antithyroid antibodies is complicated. Iodine deficiency can result in nodular goiter, where thyroid antigens are secreted from the abnormal gland, causing anti-thyroid antibodies to be present in the circulation. However, excessive iodine intake or increased iodine intake in an iodine-deficient population also increases the risk of thyroid autoimmunity, as confirmed by studies in many countries (118). Iron is indispensable for efficient iodine use and synthesis of thyroid hormone. Iron deficiency is a common symptom of hypothyroidism. It is diagnosed in around 60% of these patients (119). Khatiwada et al. noticed meaningfully lower levels of iron in hypothyroid Nepalese children and a significant correlation between anemia with hypothyroidism and iron deficiency. Anemic children tended to have higher TSH, and children with iron deficiency had much lower fT3 levels than children with sufficient amounts of iron (120). Deficiencies of iodine and iron often coexist. Numerous randomized scientific studies in populations at high risk for iron deficiency anemia and goiter came to the inference that the combined treatment with iodine and iron is better than the iodine treatment alone (121–124).

A study conducted by Peepre, Deshpande, and Choudhary (125) showed that Vitamins C, E, and turmeric are positive thyroxine modulators. Wistar rats were used in the experiment and were given Vitamin C, E, and turmeric. It was demonstrated that the application of antioxidants, i.e., Vitamin C, Vitamin E, and turmeric, greatly increased the circulating levels of T3 and T4, which could be caused by the direct participation of antioxidants on the thyroid gland or the activity of deiodinase enzyme (125).

Proper nourishment of the body is an essential element in the diet therapy of Hashimoto. Equally important is the regulation of the immune system by an anti-inflammatory diet. Observational and controlled studies have shown common nutrition deficiencies in HT patients. Experts point to the importance of a proper level of dietary fiber, protein intake, and unsaturated fatty acids, in particular the n-3 family. The study by Ruggeri et al. suggests that a low intake of animal-based foods has a protective effect on thyroid autoimmunity, and points to the positive effects of such dietary patterns on redox balance and potential oxidative stress-related disorders (126). There is a lot of debate over dietary modifications in the management of Hashimoto’s thyroiditis including elimination of gluten and lactose. According to the current state of knowledge elimination of gluten in HT should be implemented in the coexistence of celiac disease, or non-celiac gluten sensitivity syndrome. Otherwise, there is no evidence to support a gluten-free diet in HT without gluten-dependent diseases (127, 128). Up to 75.9% of the patients with HT suffer from lactose intolerance. Lactose intolerance is particularly dangerous for patients taking levothyroxine. Lactose intolerance decreases the bioavailability of the medicine and requires the use of higher doses of levothyroxine. Thus, if there is no expected reaction to the treatment with levothyroxine, tests for lactose intolerance may be considered (128) (**Table 2**). Lactose-free diet is recommended in patients with HT and lactose intolerance.

The research has shown that anatabine, an alkaloid present in Solanaceae plants, improves the mouse model of Hashimoto’s thyroiditis (129). In a randomized, multi-site study, levels of absolute serum TgAb have been significant reduction in patients treated with anatabine (130). Longer-term effects should be investigated further (101). It is well known that the lifestyle of patients with Hashimoto’s thyroiditis can play a significant role in disease management (131).

## 6 Treatment of PCOS

Treatment of PCOS can be divided into treatment of acute complaints and treatment of chronic complaints. Acute ailments include irregular periods and hirsutism, while chronic ailments are mainly treated through infertility treatment (132). The first and most common treatment for PCOS is the use of hormonal birth control pills (133). Such therapy is used to treat PCOS symptoms such as hirsutism, acne, and menstrual disorders (132, 134). These remedies are effective at restoring a normal menstrual cycle. Hormonal contraceptives containing non-androgenic progestin, which lowers ovarian secretory function, reduce overall androgen levels in the body and leads to restoration of a normal menstrual cycle (135). Excessive testosterone production in PCOS is mainly due to the increased release of luteinizing hormone by the pituitary gland and the effects of a hyperinsulinemic state on the ovaries. Contraceptives used to treat polycystic ovary syndrome stimulate an overall increase in SHBG and reduce gonadotropin synthesis. As a result, they reduce overall testosterone levels in the body by 40 to 60% (132). This also makes them effective in treating hirsutism. Studies report that third-generation oral contraceptives improve overall treatment outcomes by 33% (132). Studies show that oral contraceptives containing ethinylestradiol or progesterone are relatively safe and effective in the treatment of PCOS. A dose of 50 mg of ethinylestradiol has been shown to have a positive effect on menstrual cycle regulation in obese women. Another study conducted by Greek researchers suggests that the combination of 150mg desogestrel with 30 mg ethinylestradiol noticeably lowers overall androgen levels, reduces symptoms of hirsutism in adolescents, and restores a normal menstrual cycle. This combination did not adversely affect the body; there was no increase in body weight, and the normal lipid profile and waist-to-hip ratio were maintained (135).

Another course of treatment for PCOS is to reduce the effects of testosterone and its derivatives on the body (132). The anti-androgenic effect of certain substances such as spironolactone, is used here. It is an aldosterone antagonist that has a 67% affinity for the receptor for testosterone. By binding to it, it blocks the effects of testosterone on the body. The results of the study say that spironolactone reduces the symptoms of hirsutism by about 40% and shows efficacy in 50% of treated individuals when used as monotherapy (132). In polytherapy with contraceptives, the effectiveness of such a combination grows to 75%, and the reduction of hirsutism symptoms is increased to 45% (132). In addition, the drug has a high safety profile when used in low doses (136). The combination of spironolactone and metformin is also highly effective. In the study conducted, the study pool of patients was divided into two groups. Group one received metformin alone with a hypocaloric diet, while group two received metformin in combination with spironolactone and a hypocaloric diet. Such therapy was administered for 6 months. After 6 months, patients in both groups experienced a significant decrease in total testosterone levels and an increase in SHBG. An increase in FAI was observed in both groups, but the second group showed greater changes. DHEAS levels also decreased, but the changes have statistical value only for the second group (136). Another group of drugs used for the chronic treatment of PCOS syndrome are compounds from the insulin-sensitizing drug group. They are being studied as substances that reduce overall insulin levels in the body, which will lead to the potential restoration of normal endocrine and clinical parameters in women with PCOS. Drugs in this group include metformin and myoinositol (137).

One method of supporting the treatment of PCOS is metformin. It is used in women to increase menstruation, pregnancy rate, or ovulation rate. It is used in obese women, in women showing difficulty losing weight, or in women with a high BMI (132). Metformin’s action is based on minimizing hepatic glucose synthesis and raising peripheral sensitivity to circulating insulin (138). This compound, by reducing insulin levels, results in a decrease in the effect of insulin on theca cell proliferation, androgen synthesis, and endometrial proliferation. It affects glucogenesis in the ovaries by lowering it, which leads to a reduction in androgen synthesis in the ovaries (138). Taking metformin has been shown to increase menstrual regularity, lower overall androgen levels, and enhance ovulation. It also indirectly contributes to weight reduction, which also has a significant impact on the course of the condition (138).

Myo-inositol is a compound from the vitamin B complex group. It plays a significant role in the activation of many enzymes involved in normal glucose metabolism (132). This substance has been shown to have a high impact on normal ovarian function and oocyte quality (137). Studies show that women with limited inositol availability, abnormal inositol metabolism, or PCOS syndrome may develop insulin resistance (137). The study also found that women suffering from PCOS syndrome with insulin resistance have severely reduced myoinositol levels. The administration of Myo-inositol alone promotes ovulation and significantly lowers testosterone levels in the body (132). Administration of myo-inositol to PCOS patients has also been shown to reduce endocrine disruption and stimulate the reproductive axis (137). Myo-inositol has also shown great efficacy in the polytherapy of PCOS. In the study conducted, patients were divided into two groups. Group 1 received the OCP (estradiol/gestodene) tablet orally alone, while Group 2 received the same tablet in combination with myo-inositol. This study was conducted for 12 months. A greater reduction in the FG index was observed in polytherapy than in monotherapy. In addition, polytherapy showed a significant reduction in hyperinsulinemia and an improvement in fasting glucose levels. Such a change was not observed in monotherapy. Overall androgen levels decreased more in the group receiving the combination of OCP tablets and myo-inositol (139).

An important aspect of therapy for women with PCOS is to maintain a healthy body weight (132). It has been shown that weight reduction can positively affect ovulation in women with PCOS (132). Studies have suggested that reducing 5% of body weight has a positive effect on enhancing ovulation and fertility in women with PCOS (132). Being overweight and having an increased BMI is thought to exacerbate PCOS. Studies suggest that losing weight to normal levels reduces some of the unaddressed symptoms associated with PCOS. Introducing physical activity restores a normal metabolic profile. Women suffering from PCOS are advised to introduce regular, moderate physical activity. It is not recommended to force the body through rigorous, heavy exercise, or introduce strict diets (56).

Another group of substances used in the treatment of PCOS syndrome are antioxidants (140–145) (**Table 3**). These substances can be used in treatment alone or combination with other agents (145). Studies have shown that the use of antioxidants in PCOS therapy improves parameters describing the ratio of reactive nitrogen and oxygen species synthesis to antioxidant mechanisms, reduces the overall concentration of androgens in the body, and improves the physiological environment of the ovaries. Positive effects on the maturation process of ovarian follicles, regulation of metabolic processes related to lipid metabolism, maintenance of normal body weight, and reduction of the risk of chronic symptoms associated with the disease have also been noted (140–142, 144, 145). It has also been observed that these substances can have a positive effect on the overall well-being and mental condition of those affected by PCOS (141).

Among these substances is alpha-lipoid-acid (ALA), which belongs to the class of some of the most powerful antioxidants. Its action is based on its effect on the pyruvate dehydrogenase enzyme complex. It is characterized as a metabotropic antioxidant that affects the glucose transporter (GLUT-4) causing an increase in its expression, thereby reducing total blood glucose concentrations (141, 146–150). To confirm the efficacy of ALA use in PCOS, a study was conducted with 6 patients who had PCOS but no diabetes. In the study, ALA was administered twice daily at a dose of 600 mg. At the final stage of the study (week 16), a reduction in LDL-4 particle fractions, a reduction in triglycerides, and a significant improvement in cellular sensitivity to insulin was observed (141). Another study exploiting the potential of ALA was conducted on 45 women suffering from PCOS. These women were divided into two groups: patients receiving ALA and a group receiving a high-protein diet (20 and 25 subjects, respectively). It was observed that those who received ALA before eating breakfast had a lower rate of hyperinsulinemia than those who did not receive ALA. In the 16 patients in the group receiving ALA who suffered from the absence of menstruation after the study received it, the development of follicles was observed, and the volume of ovaries returned to normal (141).

Other substances in this group are vitamins C and E. Vitamin C acts as a cofactor in enzymatic reactions, and owes its antioxidant activity to direct reactions with superoxide radicals and by influencing the normal levels of other substances with antioxidant potential in the vitamin group (141, 151–154). Vitamin E also belongs to the antioxidant group and is involved in the activation of antioxidant enzymes present in the body’s cells. Through its activity, it prevents the peroxidation of lipids present in cell membranes (141, 155–157). Animal studies have been conducted to evaluate the effects of vitamin C on the current PCOS. The disease was induced by dehyroepiandrostendione in rodents. As a result of the study, rodents that received vitamin C showed an increase in antioxidant substances, a decrease in cytokines in the body, and a reduction in pathological cystic ovarian lesions and atretic lesions. The results also showed a reduction in the expression of genes for androgen receptors (141). Another study evaluating the effects of vitamin E involved 43 women with PCOS. The group was randomly divided into 2 subgroups. For 8 weeks, the 22-person group received 400 IU of vitamin E daily in the form of alpha-tocopheryl acetate, and the 21-person group received cellulose capsules. Upon completion, it was observed that vitamin E intake significantly improved ovarian blood flow, normalized ovulatory processes, and influenced the normal proliferation of ovarian follicles. A reduction in fat and weight was also observed (141).

Coenzyme Q10 also belongs to the antioxidant group of compounds used in the treatment of diseases. This molecule is involved in the proper process of energy supply and utilization by the body’s cells. It also plays a very important role in the proper process of aerobic cellular respiration, the synthesis of energy used by the cell (ATP), and is an essential component of a properly functioning electron transport chain (141, 158–162). A study was conducted to evaluate the antioxidant efficacy of coenzyme Q10 in women with PCOS. A group of 44 women was collected and divided into 2 subgroups (21 and 22 subjects). The 21-person group received placebo at a dose of once daily, and the 22-person group received 200mg of coenzyme Q10 once daily. The study lasted 8 weeks. At the end of the study, significant improvements in ovarian function and improved values for markers of endothelial dysfunction and markers of inflammation were observed in patients in the 22-person group (141).

Another substance with antioxidant properties is N-acetylcysteine (NAC). This substance has apoptotic effects, modulates the activity of the insulin receptor, and can affect the body’s glucose utilization and utility. In addition, this molecule can reduce the overall concentration of homocysteine in the body (142). N-acetylcysteine exhibits antioxidant properties through its ability to reduce glutathione concentrations and modulate the concentrations of other antioxidant substances (141, 163–167). In women suffering from PCOS and hyperinsulinemia, N-acetylcysteine can affect the body’s insulin sensitivity by modulating the concentration of circulating insulin in the body (142). A study of the effectiveness of N-acetylcysteine enlisted the help of 37 women with PCOS. The study was conducted for 6 weeks. During this time, the subjects were given 1.8g of N-acetylcysteine once a day; obese subjects were given a dose of 3g of NAC per day. At the end of the study, a reduction in insulin and free androgen levels was observed in those with hyperinsulinemia. An increase in the body’s cellular sensitivity to insulin was also observed (141). Another study included 60 female PCOS patients who received intracytoplasmic sperm injection. The overall group was divided into 4 subgroups: group 1 received NAC at 1800 mg per day; Group 2 received metformin at 1500 mg per day; Group 3 received a combination of NAC and metformin; and Group 4 received a placebo. The study was conducted for 6 weeks. At the end of the study, it was shown that there was a reduction in the number of oocytes with pathological structures and immature oocytes in women who received NAC. An overall increase in embryos of good quality was also observed. A decrease in insulin, leptin, and lutotropin levels was observed in the groups that received a combination of both NAC and metformin and N-acetylcysteine alone (141).

Melatonin is another substance with antioxidant potential. It is a tryptophan derivative secreted from the pineal gland that plays an important role in regulating the diurnal rhythm. It also exhibits high antioxidant activity through its ability to enter into direct reactions of a cascade nature with free radicals. In addition, it also can reduce free radicals. This process takes place by affecting the improvement of the respiratory chain function and by direct expression of the mitochondrial genes (141, 168–172). A study of the effects of melatonin on PCOS was conducted on female rodents. The female rats were 21 days old and given a single dose of testosterone of 20 mg per kilogram of body weight for 35 days to induce PCOS. During the study, female mice were given metformin alone at 500 mg per kg, melatonin at 1mg per kg, or a combination of melatonin at 2mg per kg with testosterone. The substances were administered for 36 days. As a result of the study, it was observed that the administered melatonin had a beneficial effect on the lipid profile, the percentage of fat present in the abdominal cavity, the body weight of the rats, and also the overall insulin concentration in the body. After histopathological examination, a reduction in follicles with cystic changes was observed. A reduction in neoplastic lesions within the endometrial glands and a beneficial effect on adipocyte hypertrophy were also noted (141). Another study conducted on a group of 40 patients suffering from PCOS, who were characterized by normal weight, the presence of hyperandrogenism, and irregular menstruation, showed a beneficial effect on the aforementioned conditions. Melatonin was administered to the study women for 6 months (141). A study was also conducted on a group of 58 patients. The general group was divided into two subgroups, each of which received: group 1 - melatonin 10 mg, and group 2 - placebo. Both groups received the substances for 12 weeks of the study, one hour before bedtime. The study showed a positive effect of melatonin received by group 1 subjects on insulin levels, general mental well-being, LDL indicators, and cholesterol levels (**Table 2**) (141).

**Table 2:** Therapeutic strategies of HT and PCOS.

Therapeutic strategies of HT	Therapeutic strategies of PCOS
• Thyroid hormone therapy • Therapy with glucocorticoids • Selenium supplementation • An anti inflammatory diet	• Hormonal birth control pills • Reduction of testosterone effects and its derivatives on the body • Metformin and myo inosytol • Maintaining a healthy body weight

**Table 3:** Antioxidants used in the treatment of HT and PCOS.

Antioxidants used in HT treatment	Antioxidants used in PCOS treatment
• Vitamin E and C • Selenium • Zinc • Turmeric • Iodine • Natural polyphenols and alkaloids • Inositol • L-carnitine • Probiotics	• alpha-lipoid-acid (ALA) • Vitamins C and E • Coenzyme Q10 • N-aectylcysteine (NAC) • Melatonin

## 7 Summary

In recent years, a significant increase in the simultaneous incidence of HT and PCOS in women has been observed. Destruction of the thyroid and ovaries may be caused by greater exposure to autoantibodies, and the consequence of both disorders may be insulin resistance, impaired glucose tolerance, weight gain, and infertility. Despite the knowledge in this field, the mechanism of PCOS and HT is still unclear. Studies have shown that a very important role in the pathogenesis of the coexistence of both diseases is played by chronic stress, which leads to the excessive generation of free oxygen radicals and, consequently, oxidative stress. Increased oxidative stress pathways lead to increased synthesis and release of a wide variety of immune and inflammatory molecules. In patients suffering from these diseases, there is a significantly disturbed oxidation-reduction balance. This hypothesis is confirmed by studies confirming the role of antioxidants in the prevention and treatment of these diseases. Future studies on the role of oxidative stress in the comorbidity of both diseases should further clarify the nature of these processes, identify common mechanisms involved in the course of diseases, and consider the role of antioxidants in the development of therapeutic strategies.

## Author contributions

Conceptualization, methodology, project administration: MH. Writing - original draft: GB, AD, EB, MH. Writing - review & editing: IP-C, MH. All authors contributed to the article and approved the submitted version.
